# Impact of haematocrit on the accuracy of spot blood glucose measurements in dogs using two veterinary hand-held glucometers

**DOI:** 10.3389/fvets.2025.1694081

**Published:** 2025-12-04

**Authors:** Mariona R. Garcia, Nicholas Mann, Marina Hernandez Perelló, Valentina Silic, Tim Sparks, Helen Titmarsh

**Affiliations:** 1 Wear Referrals, Bradbury, United Kingdom; 2 Waltham Petcare Science Institute, Leicester, United Kingdom

**Keywords:** anaemia, veterinary glucometers, blood glucose, dogs, critical care

## Abstract

Anaemia is a common finding in critically ill dogs, and understanding its potential impact on the accuracy of point-of-care glucometers is essential. In human medicine, it is well reported that haematocrit can affect blood glucose measurements and influence clinical outcomes when measured using certain hand-held glucometers, whereas evidence in veterinary medicine is still scarce. This prospective case–control study included 72 client-owned dogs, 39 of which were anaemic. Haematocrit values were used to classify dogs into anaemic and non-anaemic categories. Whole blood glucose concentrations were measured using two new models of veterinary hand-held glucometers (G-PET PLUS^®^ and AlphaTrak3^®^) and compared with the serum blood glucose measurements obtained with an automated wet biochemistry analyser. Agreement between the point-of-care blood glucose analysers and the reference method, as well as the effects of haematocrit on measurement accuracy, was assessed. Both hand-held glucometers had a significant positive correlation with the wet biochemistry analyser (AlphaTrak3^®^: r = 0.664; 95% CI 0.502, 0.78; G-PET PLUS^®^: r = 0.769; 95% CI 0.643, 0.855). However, when comparing the overall blood glucose results, there was a significant negative percentage bias (*p* < 0.001) for both devices. Haematocrit reductions did not affect the degree of bias for AlphaTrak3^®^, and for the G-PET PLUS^®^, bias was also present in control patients and in patients with a mild haematocrit reduction. Despite these statistical differences, clinical impact assessments of the results via specialist clinician questionnaires and Parkes error grid analysis showed no change in clinical decision-making or patient outcome, supporting the cautious use of these devices in anaemic but normoglycaemic dogs when testing whole blood EDTA samples.

## 1 Introduction

Hand-held, point-of-care blood glucose analysers, which use whole blood, are commonly used in veterinary practice. In many veterinary clinics, these monitors will be used for critically ill and emergency patients when results are required for instantaneous decision-making. These monitors are attractive in this setting because of their rapid turnaround time and requirement for minimal sample volumes, although their use is not recommended by some manufacturers for critically ill patients (1). Clinicians also have access to automated biochemistry analysers, either external or in-clinic laboratories that measure serum or plasma blood glucose. Laboratory-based analysers, which commonly use hexokinase or glucose oxidase reactions, are considered the reference methods to evaluate blood glucose (2–4). However, despite their accuracy, these require larger sample volumes, separation of whole blood samples to obtain plasma or serum, and slower turnaround time, making them less convenient in comparison with handheld glucometers in situations where rapid decision-making is imperative or only small samples can be collected. Despite the advantages of hand-held glucometers, it is well reported in critically ill human patients that the haematocrit of blood samples impacts the accuracy of blood glucose measurements obtained by hand-held glucometers (5, 6). Therefore, guidance states that some of these analysers should not be used to direct treatment aiming to adjust glycaemic control in human patients with an abnormal haematocrit (7, 8). Anaemia is a common laboratory finding in veterinary practice, estimated to be present in approximately 30% of critically ill dogs on admission (9, 10), who may also have concomitant blood glucose changes that may affect clinical decision-making or prognosis. It is therefore important to understand how anaemia and different severities of anaemia impact the accuracy of blood glucose measurements assessed by point-of-care glucometers.

Point-of-care hand-held blood glucose analysers utilise whole blood samples, where glucose is found within both cells and in the plasma (1, 11). The results displayed are typically a calculated value that approximates the amount of glucose expected to be found in plasma. Plasma contains proportionally more water than red blood cells, and the total water content of whole blood will vary according to haematocrit. Therefore, the accuracy of calculated values may be influenced by the sample haematocrit (1, 2). A low haematocrit could affect the accuracy of hand-held glucometers through two main mechanisms. First, as a consequence of a low haematocrit, the diffusion rate of plasma into the reagent pad may be increased. Second, because of their built-in conversion factors to convert whole blood glucose measurements into plasma-equivalent values (5, 6, 12, 13).

In 2016, the American Society for Veterinary Clinical Pathology (ASVCP), in guidelines regarding the quality assurance for portable blood glucose meters, stated that erroneous results could be expected on whole blood glucose measurements when haematocrits fall outside 40–50% (2). Previous studies have assessed the impact of haematocrit in canine samples on readings obtained using hand-held glucometers validated for either use in people or specifically for veterinary use. Paul et al. (14) investigated the impact of haematocrit on the accuracy of point-of-care blood glucose readings. They reported that both high and low haematocrit impacted the accuracy of hand-held blood glucose monitors. However, of the 184 dogs sampled, 139 were Greyhounds, a breed that typically has haematocrits greater than reference intervals for other dog breeds (14). There were 17 dogs in the study, which were anaemic, most of which were only mildly anaemic; however, in these anaemic dogs, a veterinary-specific glucometer tended to overestimate blood glucose compared to the reference method (14). Moreover, Greyhounds are a poor representation of the general canine population due to their well-known physiologic differences affecting glucose and red blood cell-related laboratory results (15). Johson et al. (16) also compared glucose measurements between a point-of-care glucometer validated in humans, a point-of-care glucometer validated for veterinary use, and a reference plasma biochemistry analyser. Their results found no exact agreement between the portable glucose meters and the chemistry analysers, with the discordance suspected to be exacerbated by higher and lower haematocrits. However, haematocrits were not recorded for all the study population and only ranged between 25.9 and 59.2% (16). Similarly, Suchowersky et al. (17) were able to determine a median variation of 1.5 mmol/L (27 mg/dL) in glucose measurements between a portable blood glucose meter (AlphaTrak 2^®^; Zoetis) and a biochemical analyser in anaemic samples. However, only 13 anaemic samples were analysed, with a mean haematocrit of 34% (17). Lane et al. (13) diluted packed red cells collected from dogs with plasma to create blood samples with varying haematocrits. This was used to develop a predictive model to correct blood glucose concentrations for patients with haemoconcentrated and haemodiluted samples based on a single type of veterinary-validated point-of-care glucometer (AlphaTrak 2^®^; Zoetis). The model was then applied to a population of 30 canine patients with packed red blood cell counts of 12–72%. Uncorrected haemodiluted samples had higher blood glucose concentrations when measured using the hand-held glucometer, whilst lower blood glucose concentrations were seen in haemoconcentrated samples compared to the reference analyser (13). This study, like others, indicated that blood glucose was overestimated in anaemic patients; however, it was performed using a veterinary glucometer, which has since been replaced by the manufacturer with a new model.

The current study aims to determine the impact of different haematocrit levels on blood glucose measurements obtained using two new models of commonly hand-held glucometers validated for use in canine patients currently available on the veterinary market. If a bias was observed when measuring blood glucose using these hand-held glucometers in patients with a reduced haematocrit, a second objective was to determine its clinical significance. The blood glucose results will be compared with a serum wet chemistry analyser, considered the reference analyser (2–4).

We hypothesised that, at lower haematocrits, both hand-held blood glucose analysers would have overestimated the serum glucose readings.

## 2 Materials and methods

This is a prospective study conducted at a private referral centre based in the United Kingdom, between January and March 2025. Ethical approval was obtained from the RCVS Ethics Review Panel on the 1st of November 2024 (reference 2024-071-Rossell).

### 2.1 Case selection

Seventy-three client-owned dogs presenting to the hospital were enrolled, and written consent for inclusion in the study was obtained from all owners. Inclusion criteria required that these dogs have blood tests for diagnostic or treatment purposes unrelated to this study. Dogs were considered for inclusion if the primary clinician responsible for the case requested blood samples to be collected in plain serum tubes and an ethylenediaminetetraacetic acid (EDTA) tube, and a haematocrit result was available. Blood samples were never collected for the sole purpose of this study, and, unless already requested as part of the patient investigations, blood glucose measurements using the AlphaTrak3^®^ and G-PET PLUS^®^ were performed on residual blood samples remaining after all the tests ordered by the lead clinician were completed.

Haematology profiles were used to determine the patient’s haematocrit. Depending on the haematocrit obtained, patients were classified with mild haematocrit reduction (haematocrit between 30 and 40%), moderate (between 20% and 29%), and severe (less than or equal to 19%). This is similar to categories used by other researchers studying anaemia (9, 14, 18, 19).

Further requirements for inclusion criteria were having sufficient excess blood in the EDTA and plain blood collection tubes after all tests requested by the primary clinician had been completed, the sample tubes had to be labelled with the blood collection time, and the blood glucose measurements had to be run within an hour of collection.

Control patients or dogs with haematocrit reductions were excluded if there was not enough residual blood for analysis, if blood glucose could not be measured consecutively and within 1 h of collection in all the analysers, or if owners had not consented to the use of their pets’ data in the study.

### 2.2 Blood collection

Blood samples were collected by venipuncture of a peripheral vein (e.g., jugular, cephalic, and saphenous veins) and transferred into the standard size of 1.3 mL of EDTA samples tubes (International Scientific Supplies Ltd., UK) and 1.3 mL plain serum tubes (Sarstedt, Germany). The tubes were labelled with the patient’s name, date, and time of collection. The EDTA tubes were gently inverted immediately after blood collection. The plain serum tube was centrifuged at 1200 rpm for 3 min in the in-house laboratory, and the serum was harvested in a plain Eppendorf. Samples were not refrigerated prior to analysis.

### 2.3 Data collection

The age, breed, sex, and neuter status of each dog were recorded alongside the haematocrit, blood glucose measurements for each analyser, and time of blood collection, as well as the time the blood glucose was measured.

### 2.4 Sample analysis

All samples were run in the in-house laboratory by two trained, dedicated laboratory technicians.

Haematology profiles were run on an IDEXX ProCyte Dx Haematology Analyser^®^ (IDEXX Laboratories, Inc., United States), which used a combination of laser flow cytometry, optical fluorescence, and laminar flow impedance, and a minimum of 500 μL of EDTA blood sample was needed. Blood glucose was measured on serum samples using the wet automated biochemistry analyser Woodley InSight DB analyser^®^ (Quantum Vet Diagnostics, Woodley Equipment Company Ltd., UK) via the enzymatic hexokinase reaction. The analyser required 180 μL to run this test. The same EDTA whole blood sample used for the haematology profile was used to measure whole blood glucose on the two new models of hand-held glucometers AlphaTrak 3^®^ (Zoetis, United States) and G-PET PLUS^®^ (Woodley Equipment Company Ltd., UK). Both measure glucose using a glucose dehydrogenase reaction and require 0.3 μL and 0.7 μL of sample, respectively (1, 11). Both manufacturers stated that the use of the hand-held glucometers was validated in whole blood capillary or venous samples as well as in heparin tubes. AlphaTrak 3^®^ was the only one reported by the manufacturer to be validated for use with EDTA whole blood samples and blood glucose, although, unlike in our study, the manufacturer stated glucose should be measured within 10 min of collection (1, 11). All samples in the study were analysed up to 1 h after collection. To ensure time consistency between the blood glucose readings of whole blood and serum samples, the latter was centrifuged and separated immediately before analysis, and analysis using each handheld glucometer and the serum biochemistry analyser was performed concurrently.

The quality control of the ProCyte Dx Haematology Analyser^®^ was performed weekly by a trained, dedicated laboratory technician and quality assured once a month by an external provider (SRUC Veterinary & Laboratory Services, Pentlands Science Park, Bush Loan, Penicuik, Midlothian, EH26 0PZ, United Kingdom, scheme number 10143534). The wet chemistry analyser Woodley InSight DB analyser^®^ also had daily quality controls and monthly external quality assurance controls from the same provider. Both handheld glucometers underwent monthly internal quality control and were calibrated every time a new vial of test strips was opened, in accordance with the manufacturer’s instructions (1, 11).

### 2.5 Clinical impact

Three ECVIM-CA small animal internal medicine diplomates working at the same referral centre were asked to classify all the blood glucose results for the three analysers as being normoglycaemia, hyperglycaemia, and hypoglycaemia. The clinicians were blinded to the analyser and reference ranges. They also had to indicate whether any of the results would have prompted a clinical action (e.g., rechecking the result, performing further diagnostic tests, or adjusting treatment). The haematocrit of the sample, the analysers used, and the respective reference ranges of the analysers were concealed during the assessment.

Parkes error grids comparing serum glucose and point-of-care glucometer measurements were created using the Ega package in R 4.3.3 (20).

### 2.6 Statistical analysis

Sample size estimates derived from R routines, based on a power of 0.80, a significance level of 0.05, and a medium effect size, sensu Cohen (20), suggested a minimum sample size of 67.

Information on age, sex, breed, neutering status, and the time between collection and processing of the blood sample was summarised by frequency and percentage, or as mean and standard deviation, as appropriate. The serum blood glucose measurement obtained with the automated wet biochemistry analyser was considered the ‘gold standard’ control measurement over which the results of hand-held whole blood glucose samples were compared.

Bias between each glucometer and the gold standard wet biochemistry was calculated as the difference between the two methods (as a percentage of wet biochemistry values), with negative values underestimating glucose compared to the wet biochemistry values. Overall bias for each method was evaluated using Wilcoxon signed-rank tests against a hypothesised median value of zero.

Four haematocrit reduction levels were established (severe 19% or below; moderate, from 20 to 29%; mild 30 to 40%; control, above 40%). Differences in bias for each method between the haematocrit categories were evaluated using Kruskal–Wallis tests adjusted for ties.

Bias in individual haematocrit classes for AlphaTrak3^®^ and G-PET PLUS^®^ was compared to a median of zero using Wilcoxon signed-rank tests. Associations between variables were assessed using Spearman’s rank correlation. Analysis was undertaken using commercial software (Minitab 21), and significance was taken as a *p*-value <0.05.

## 3 Results

A total of 80 blood samples were obtained from 73 client-owned dogs. One sample was excluded, as the sample was run 63 min after the samples were collected. Seven of these dogs were included twice, using blood samples collected on separate days, leaving 79 samples collected from 72 client-owned dogs in the study. These repeat cases required repeated blood sampling for monitoring and treatment of chronic conditions, including chronic kidney disease, chemotherapy administration, chronic enteropathy, and primary hyperparathyroidism.

The haematocrit of the samples ranged from 10 to 58%. Of the 79 blood glucose measurements, 8% (6/79) corresponded to patients with severe haematocrit reduction (0–19%), 14% (11/79) of dogs had a moderate haematocrit reduction (20–29%) and 25/79 had a mild reduction (haematocrit 30–40%). The remaining 40 samples were considered controls (haematocrit above 40%).

The median age for the dogs was 7 years (range: 6 months to 14 years). Breeds included were 1 Akita, 2 Australian Labradoodle, 1 Bedlington Terrier, 1 Bernese mountain dog, 1 Border Collie, 3 Border Terriers, 1 Boston Terrier, 2 Cavalier King Charles Spaniel, 2 Chihuahuas, 7 Cockapoos, 9 Cocker Spaniels, 11 crossbreeds, 2 Dachshunds, 2 Dalmatians, 1 Doberman, 1 English Bull Terrier, 4 French Bulldogs, 1 German Pointer, 2 German Shepherds, 2 Goldendoodles, 2 Jack Russell Terriers, 10 Labrador Retrievers, 1 Leonberger, 1 Maltese, 1 Old English Sheepdog, 1 Rhodesian Ridgeback, and 1 Rottweiler. Three of the females were entire, and 24 were neutered. For the male dogs, 23 were entire and 22 were neutered.

### 3.1 Comparative analysis

**Figure 1:**
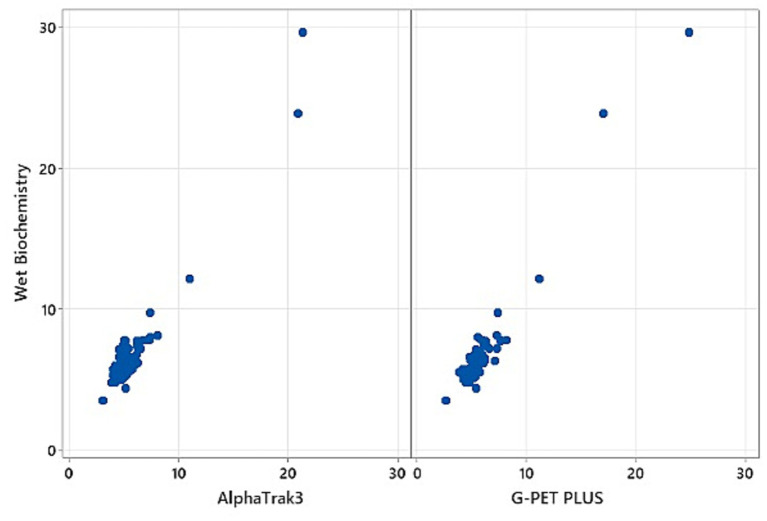
Scatterplot of the results of the AlphaTrak3^®^ and G-PET PLUS^®^ against the wet biochemistry analyser.

Both hand-held glucometers showed a significant positive association with the wet biochemistry analyser results. The AlphaTrak3^®^ demonstrated a positive moderate correlation, whilst the G-PET PLUS^®^ showed a stronger positive correlation (r_s_ = 0.664 and r_s_ = 0.769, respectively; both *p* < 0.001; Figure 1). However, this is unlikely to be of clinical significance, as the 95% CIs were 0.50–0.78 and 0.64–0.85, respectively, with a considerable overlap.

When all 79 samples were considered together, the AlphaTrak3^®^ and G-PET PLUS^®^ both significantly underestimated the blood glucose results compared to the bench biochemistry analyser (*p* < 0.001). Table 1 presents the range of blood glucose readings between the different analysers as well as the percentage difference between each portable hand-held glucometer and the wet biochemistry.

**Table 1:** Glucose concentrations measured using two hand-held glucometers (AlphaTrak3^®^ and G-PET PLUS^®^) and a wet biochemistry analyser, including the median percentage difference and its 95% confidence interval between each of the glucometers and the wet biochemistry analyser.

Analyser	Glucose concentration (mmol/L)		Median % difference	95% CI of median difference	*p*-value
	Median	Range			
AlphaTrak3^®^	5.2 (93.6 mg/dL)	3.1–29.4 (55.8–529.2 mg/dL)	−14.1%	(−16.4, −11.7)	<0.001
G-PET PLUS^®^	5.4 (97.2 mg/dL)	2.7–24.8 (48.6–446.4 mg/dL)	−11.3%	(−13.5, −9.0)	<0.001
Wet biochemistry analyser	6.1 (109.8 mg/dL)	3.5–29.6 (63–532.8 mg/dL)	-	-	-

**Figure 2:**
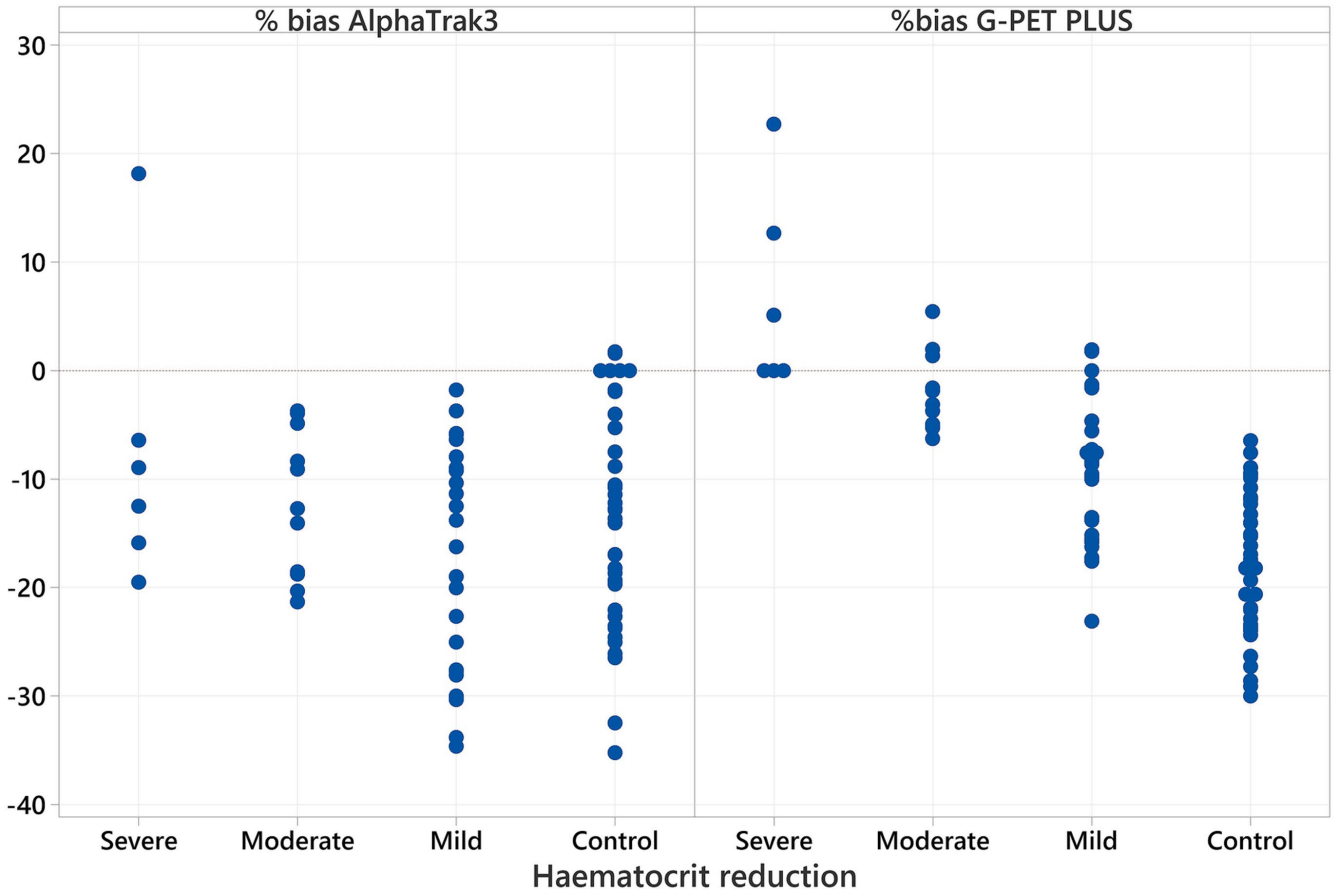
Percentage bias for the AlphaTrak3^®^ and G-PET PLUS^®^ glucometers. The horizontal line at 0% indicates perfect agreement with the gold standard wet biochemistry analyser. Values above this line represent overestimation of glucose levels, whereas values below indicate underestimation for each glucometer and haematocrit group.

**Figure 3:**
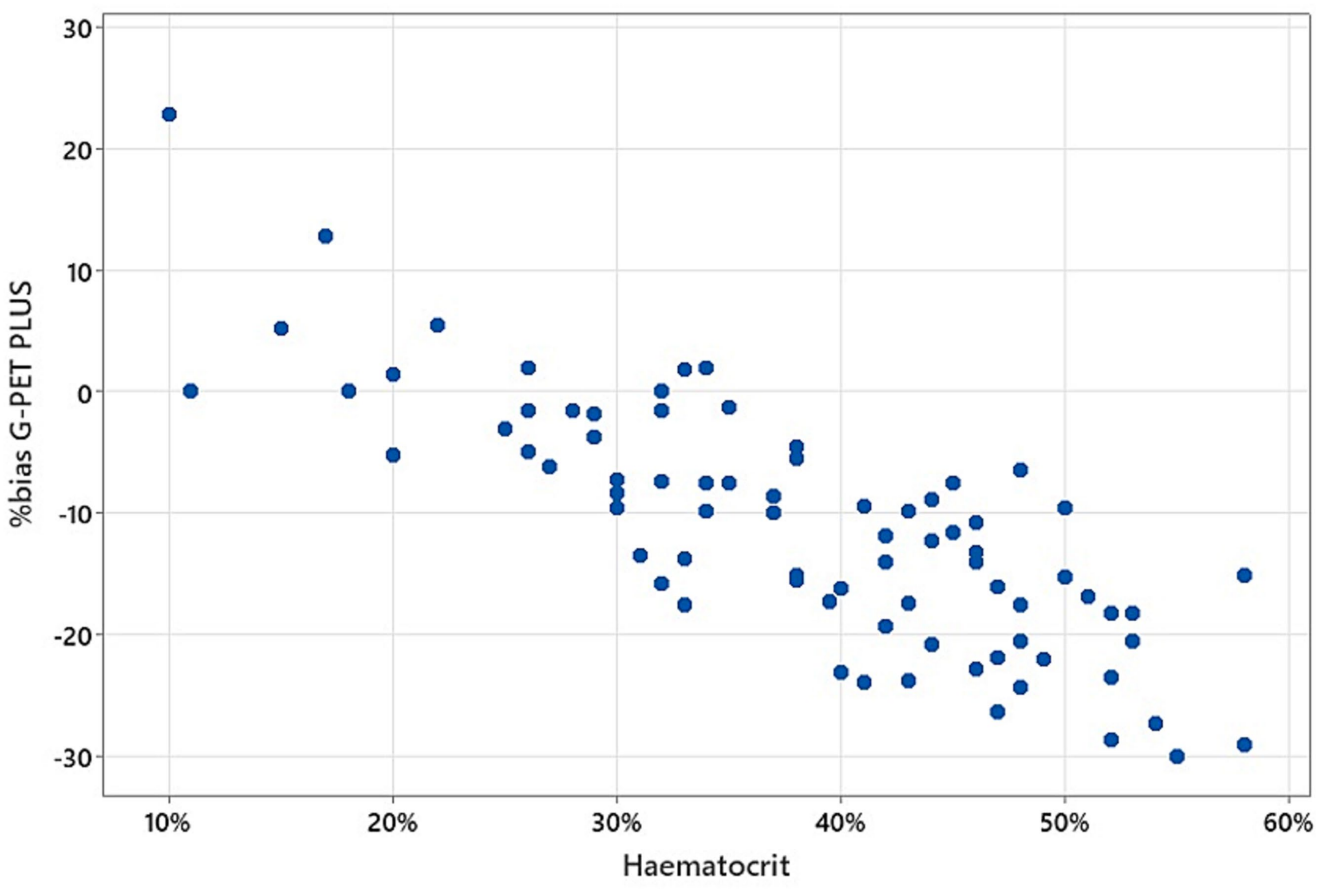
Scatterplot demonstrates the negative correlation between the percentage bias of the blood glucose readings obtained using the *G-PET PLUS*^®^ glucometer and those from the wet biochemistry analyser. A 0% bias indicates complete agreement between the two analysers.

For AlphaTrak3^®^, there was no significant difference in percentage bias of blood glucose measurements across all haematocrit categories (*p* = 0.447) nor a correlation with haematocrit (r_s_ = −0.066, *p* = 0.561). However, the percentage bias within each haematocrit category showed a statistically significant underestimation in the control, mild, and moderate haematocrit reduction groups (Table 2; Figure 2). The percentage bias in the results obtained from G-PET PLUS^®^ differed significantly between haematocrit categories (*p* < 0.001). A strong, significant negative correlation (r_s_ = −0.794, *p* < 0.001) was evident between haematocrit and percentage bias in blood glucose readings for the G-PET PLUS^®^ glucometer (Figure 3). However, the percentage bias was only significant in the control and patients with a mild haematocrit reduction. Interestingly, despite not being statistically significant, the G-PET PLUS^®^ glucometer tended to overestimate the blood glucose results in patients with severely decreased haematocrit values (Table 2; Figure 2).

**Table 2:** Median bias and median percentage bias across haematocrit reduction categories using the AlphaTrak3^®^ and G-PET PLUS^®^.

	AlphaTrak3^®^ glucometer			G-PET PLUS^®^ glucometer		
Haematocrit reduction category	Median (range) bias (mmol/L)	Median (range) percentage bias	*p*-value for the median percentage bias	Median (range) bias (mmol/L)	Median (range) percentage bias	*p*-value for the median percentage bias
Severe	−0.5 (−1.5 to 0.8)	−10.7 (−19.5 to 0.8)	0.295	0.2 (0 to 1)	2.6 (0 to 18.2)	0.181
Moderate	−0.8 (−1.3 to −0.2)	−12.7 (−21.3 to −0.2)	0.004	−0.1 (−0.3 to 0.3)	−1.9 (−4.9 to −3.7)	0.142
Mild	−1.1 (−8.2 to −0.1)	−13.8 (−34.6 to −0.1)	<0.001	−0.6 (−4.8 to 0.1)	−8.6 (−15.3 to −1.8)	<0.001
Control	−0.9 (−2.9 to 0.1)	−13.6 (−35.2 to 0.1)	<0.001	−1.1 (−6.8 to −0.4)	−17.5 (−23.1 to −1.8)	<0.001

Spearman’s correlation test also evaluated the potential effects on the percentage bias for each glucometer of patient age, delay between sample collection and analysis, and the magnitude of blood glucose on serum samples. The results demonstrated a statistically significant negative correlation between the percentage bias of both glucometers and the wet biochemistry blood glucose results, with a greater percentage bias with higher serum blood glucose results (r_s_ = −0.334, *p* = 0.003 for the AlphaTrak3^®^ and r_s_ = −0.321, *p* = 0.004 for the G-PET PLUS^®^).

**Figure 4:**
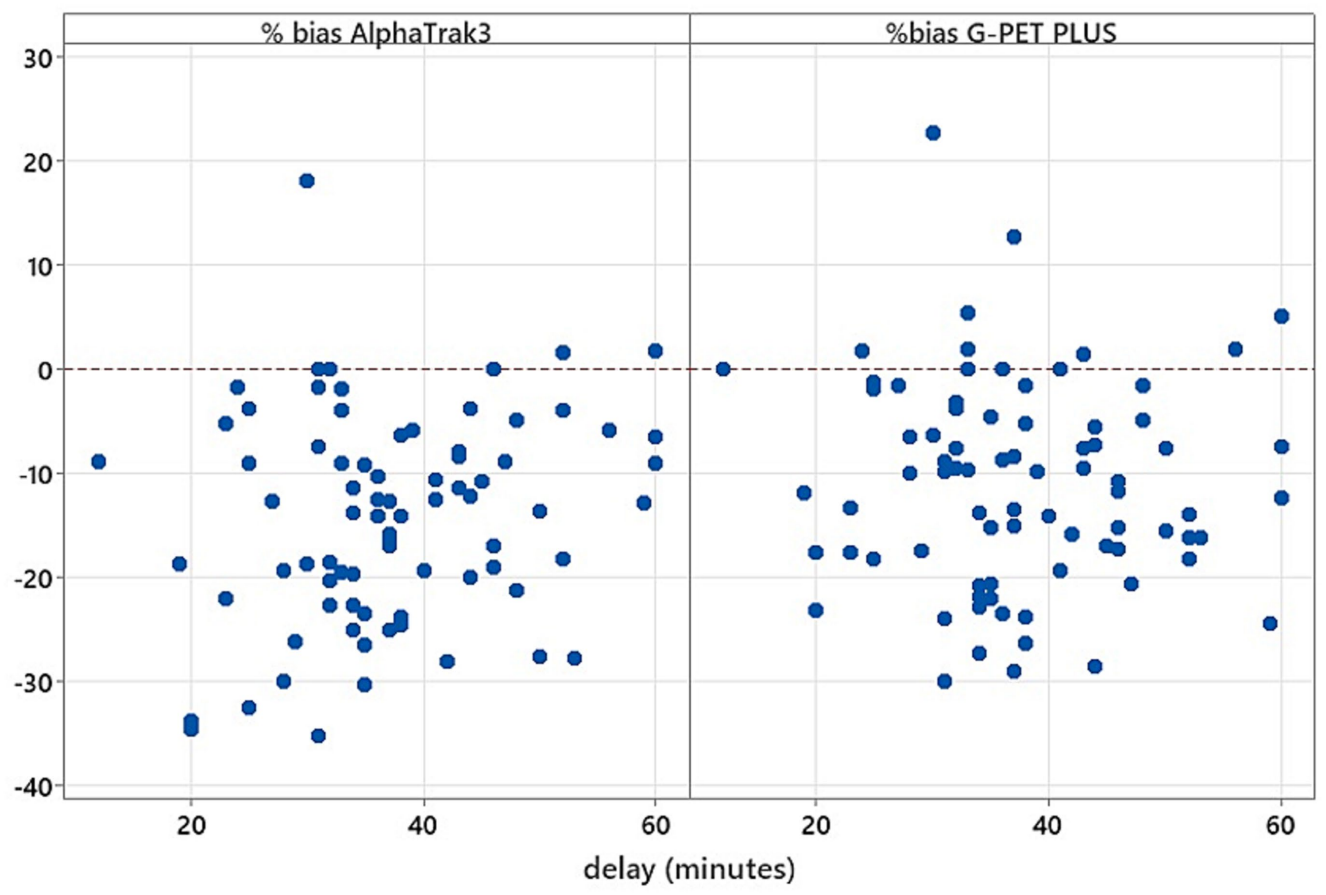
Scatterplot representation of the correlation between each glucometer percentage bias and the time from sampling to analysis. The horizontal 0 line represents perfect agreement between the glucometer and wet biochemistry. Results above the 0 line are an overestimation of the blood glucose result, and results below are an underestimation.

The median time between sample collection and analysis was 36 min (range 12 to 60 min). There was no statistically significant correlation between the percentage of bias for the AlphaTrak3^®^ (*p* = 0.114) or G-PET PLUS^®^ (*p* = 0.544) and the time to test within 60 min of collection (Figure 4). There was no significant correlation between age and percentage bias.

### 3.2 Clinical impact

The reference range for blood glucose on the wet biochemistry analyser was 4–8 mmol/L. Based on this range, one sample was identified as hypoglycaemic (3.5 mmol/L or 63 mg/dL) and five as hyperglycaemic [median of 12.1 mmol/L (217.8 mg/dL), range 8.1–29.6 mmol/L (145.8–532.8 mg/dL)]. For the AlphaTrak3^®^, the reference range was wider, 3.6–13.9 mmol/L (64.8–250.2 mg/dL) (1). Using this monitor-specific reference range, the same hypoglycaemic case was identified (3.1 mmol/L or 55.8 mg/dL). However, only two of the five hyperglycaemic samples identified by the wet biochemistry analyser were also classified as hyperglycaemic by the AlphaTrak3^®^ [21.4 and 20.9 mmol/L (385.2–376.2 mg/dL)]. The G-PET PLUS^®^ had a narrower reference range, between 4.4 and 6.6 mmol/L (79.2–118.8 mg/dL) (11). With this analyser, 5 results would had been classified as hypoglycaemic [median of 4.2 mmol/L (75.6 mg/dL), range 2.7–4.3 mmol/L (48.6–77.4 mg/dL)], only agreeing with 1/5 with the reference analyser, and 10 as hyperglycaemic [median of 7.7 mmol/L (138.6 mg/dL), range 7.1–24.8 mmol/L (127.8–446.4 mg/dL)], failing to coincide in 50% (5/10) cases with the wet biochemistry analyser.

When provided with blood glucose results of each analyser, the three ECVIM-CA specialists showed inter-clinician variability as to what cutoff blood glucose level they considered to be hypo or hyperglycaemic. For every result reported as abnormal for each of the specialists, would have triggered a change in diagnostic or treatment plan, meaning the actions taken by each clinician may have varied. However, despite the differences in agreement between clinicians, there was little or no intra-clinician variability in how the clinicians assessed the same patient as being hyper, hypo or normoglycemic. Clinician A would have made a different clinical action in 12 cases when using the AlphaTrak3^®^ analyser, whereas clinicians B and C would only have changed their clinical plan once when using this hand-held analyser, compared to the reference method. Using the G-PET PLUS^®^ clinical decisions would have differed in five cases for clinical A, one case for clinical B, and three cases for clinical C, relative to the wet biochemistry results.

Human quality standard guidelines and veterinary-specific recommendations were applied to the results of our samples to assess accuracy and clinical impact. A total allowable error is a guideline about how much a measured value can vary from the true measurement before it adversely affects clinical decision-making. The ASVCP guidelines for quality assurance for portable blood glucose meters used in veterinary medicine declare that performance testing should be performed on hand-held glucometers to ensure that repeated results from the same patient fall within the allowable total error of 10% for values below the reference interval or 20% for values within and above the reference interval (2, 21). Although we did not perform repeated measurements, if we were to apply a similar allowable error of 10% for hypoglycaemic results and 20% for normoglycemic values for patients with a haematocrit below 40%, 31 out of 39 (79.5%) values obtained AlphaTrak3^®^ and 38 out of 39 (97.8%) values for the G-PET PLUS^®^ were within the total allowable error when compared to the reference method.

**Figure 5:**
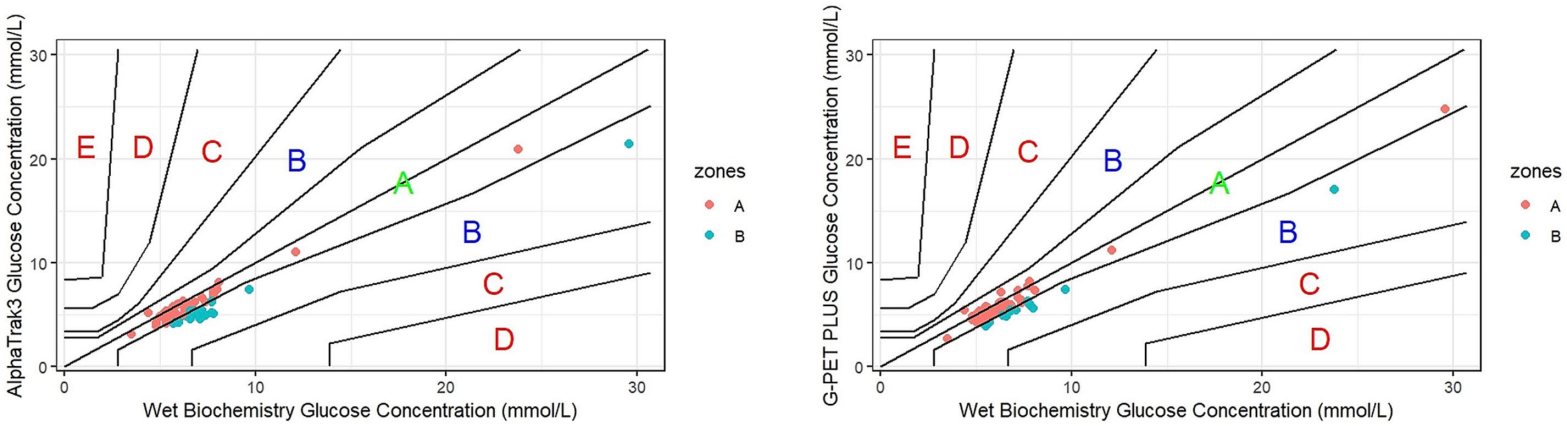
Parkes error grid analysis for blood glucose concentrations comparing measurements by the portable blood glucose monitor and reference wet biochemistry.

The Parkes error grid is a tool originally developed for human medicine to evaluate the potential clinical consequences of discrepancies between blood glucose results obtained with portable glucometers and the reference analyser in diabetic patients (22). However, the same tool had also been previously applied to dogs and cats (16, 23). The Parkes error grid defines 5 risk zones: A, clinically accurate measurements with no effect on clinical action; B, altered clinical action but little or no effect on clinical outcome; C, altered clinical action likely to affect clinical outcome; D, altered clinical action that could have a significant clinical risk; and E, altered clinical action that could have dangerous consequences for the patient (22). ISO guidelines for glucometers for human use advise that, in consensus error grid analysis, at least 99% of the results have to be within zones A and B (24). Parkes error grids (Figure 5; Table 3) were performed for both portable glucometers, and the results were compared with the gold standard wet biochemistry analyser. All blood glucose readings for the AlphaTrak3^®^ and G-PET PLUS^®^ fell within the A and B zones, having little or no effect on clinical outcome. 57/79 (72.15%) for the AlphaTrak3^®^ and 63/79 (79.75%) for the G-PET PLUS^®^ fell in the A zone. All the blood glucose results measured using the G-PET PLUS^®^ glucometer for the severe and moderate haematocrit reduction categories fell in the A zone, whilst 1/6 (16.66%) and 2/11 (18.18%), respectively, fell in zone B when measured with the AlphaTrak3^®^. Similar results were observed for the mildly decreased haematocrit category; 1/25 (4%) cases fell in the B zone for the G-PET PLUS^®^ and 8/25 (32%) for the AlphaTrak3^®^.

**Table 3:** Total and percentage of cases and percentage falling in the A and B zones for the Parkes error grid for both handheld glucometers.

Category	Total of blood glucose results	AlphaTrak3^®^		G-PET PLUS^®^	
		Zone A	Zone B	Zone A	Zone B
Severe haematocrit reduction	6	5 83.33%	1 16.67%	6 100%	0
Moderate haematocrit reduction	11	9 81.82%	2 18.18%	11 100%	0
Mild haematocrit reduction	25	17 68%	8 32%	24 96%	1 4%
Controls	37	26 70.27%	11 29.73%	22 59.46%	15 40.54%

## 4 Discussion

In human medicine, the impact of anaemia on blood glucose results obtained using portable glucometers is well documented. Similar studies have been performed in veterinary medicine with similar results showing a positive bias at lower haematocrit levels and a negative bias at higher haematocrit levels. However, most of these veterinary studies have only included a small number of blood glucose results or patients with a mild degree of anaemia (13, 16, 17). Conversely, in our study, we found the bias of the glucometers was not adversely affected by sample haematocrit, with greater agreement between glucometers and reference analysers for samples with lower haematocrits. Importantly, our results differed from previous studies evaluating veterinary point-of-care glucometers in anaemic patients, which reported higher levels of inaccuracy with low haematocrits (13, 14).

Both glucometers evaluated in this study have been validated for use on a wide range of haematocrits, 15–65% for the AlphaTrak3^®^ and 20–60% for the G-PET PLUS^®^ (1, 11). We have tested samples from patients with a greater degree of haematocrit variation than was validated by the manufacturers. When used in our clinic, as described in this study, both the G-PET PLUS^®^ and the AlphaTrak3^®^ glucometers had a good correlation with the wet biochemistry analyser. The mean percentage bias for both analysers across all the haematocrits included in this study was within 20% of the reference method. In anaemic patients, the average bias from the reference method was-5.21% and-0.82 mmol/L (−14.8 mg/dL) for the AlphaTrak3^®^, and −2.33% and-0.17 mmol/L (−3.1 mg/dL) for the G-PET PLUS^®^ (Table 2). The AlphaTrak3^®^ analyser had an overall underestimation of blood glucose results across all haematocrit categories, although the bias was not significant for dogs with severe haematocrit reductions. However, when haematocrit was considered as a single variable, the degree of reduction did not impact the degree of bias, suggesting the AlphaTrak3^®^ performed similarly in patients with different haematocrits. In comparison, whilst the G-PET PLUS^®^ blood glucose results also had a negative correlation with the wet biochemistry when haematocrits were equal to or above 30%, there was no significant bias when patients with moderate to severe haematocrit reductions were considered, although there was a statistically non-significant trend towards a positive bias in glucose readings for patients with moderate-to-severe haematocrit reductions. This finding is also different from other previous studies assessing the accuracy of hand-held glucometers on a different range of haematocrits, which showed more accuracy at normal or high haematocrits (13, 14, 16). Overall, the median bias in glucose concentrations for anaemic patients between −0.82 and −0.17 mmol/L (−14.8 and −3.1 mg/dL) for each glucometer compares more favourably than other studies where greater differences were reported. For example, Suchowersky et al. (17) reported median differences of 1.5 mmoL/L between a handheld glucometer and their gold standard method (17).

We also attempted to determine the clinical significance of the bias demonstrated by the glucometers used in the study when measuring blood glucose in patients with reduced haematocrits. Notably, the results obtained did not adversely impact the decision-making of the questioned clinicians in most cases, and all the values fell within the A and B zones on the Parkes error grid analysis. We suggest that whilst clinicians must be mindful of the manufacturer’s guidelines, which have not validated the use of these glucometers in patients with a marked reduction in haematocrit, these handheld glucometers may still be used to assess the blood glucose concentrations of anaemic patients where instantaneous results are needed, pending serum blood glucose results. Whilst both monitors performed acceptably in the majority of cases, and in no instance would the results obtained have led to treatment decisions likely to cause harm, as assessed by the Park’s error grid, care must be taken when applying our results to the typical use of veterinary hand-held blood glucometers.

Our study has some limitations. Ethical approval for our study was given to perform blood glucose measurements on residual venous blood samples. Sampling via pinpricks, which is often, but not exclusively, used with hand-held glucometers, was not possible in this study. However, it is important to note that pin-prick samples measure blood glucose in capillary blood. Several studies in human medicine have identified several factors that contribute to variation between blood glucose concentrations in capillary, venous, and arterial blood (25–28). The main parameters accounting for these differences were the fasting status, partial oxygen pressure, and tissue perfusion. However, the veterinary literature comparing capillary and venous blood glucose results found no clinically significant differences between glucose measurements (29–31). By using venous samples for both serum (where pinprick samples cannot be used for analysis) and whole blood glucose readings, we tried to avoid differences due to collection site, but this may not reflect typical use in some veterinary hospitals. However, both point-of-care glucometers used in this study have been validated for use with capillary and venous blood samples (1, 11).

Another key limitation of the study was that when using hand-held glucometers, manufacturers recommend measurements to be performed immediately or within 10 min of collection for EDTA anticoagulated blood samples in the case of the AlphaTrak3^®^. Therefore, immediate blood glucose determination would have better reflected the real clinical use of point-of-care analysers. As it is known that when serum or plasma is in contact with blood cells, glucose concentrations will decrease due to glycolysis, and it has been recommended for canine samples that at *“25 °C, serum-clot contact time should not exceed 1 h”* (32). Studies have shown that glucose concentrations in serum and EDTA plasma are not significantly different when samples are separated approximately 30 min after collection (33). Therefore, to avoid differences in blood glucose measurements from serum and whole blood occurring due to differing contact time between red cells and serum/plasma fractions of the samples, serum samples were tested immediately after centrifugation, and anti-coagulated whole blood samples were tested concurrently. Furthermore, to assess the potential impact of delay between sample collection and glucose measurement, this variable was assessed to determine whether it impacted the difference between serum and point-of-care glucometer readings. Despite the delay in testing of an average of 38 min, no significant correlation was found between the glucometer bias and time of testing within 60 min of blood collection. However, clinicians should still be mindful that blood glucose readings should be obtained as soon as possible to avoid the impact of glycolysis on the accuracy of the glucose results in real-life, clinician situations.

An additional consideration was that, whilst the AlphaTrak3^®^ manufacturers described its use with EDTA whole blood samples, this was not the case for the G-PET PLUS^®^ glucometer (1, 11). However, our results showed that when using EDTA whole blood samples, blood glucose concentrations measured by the G-PET PLUS^®^ correlated with serum glucose measurements, and the variation between serum and anti-coagulated whole blood was on average within the −20% variation considered to be an acceptable degree of bias for these types of analysers. These results were consistent with previous studies that demonstrated similar blood glucose concentrations between blood collected in plain (no anticoagulant) or EDTA tubes (2, 34). These findings also support the option to measure blood glucose using EDTA samples if the patient is not amenable to pinpricks, a serum sample is unavailable for the bench biochemistry analyser, when only limited blood samples can be taken, or when cost constraints prevent other testing options.

There were also several other limitations to our study. Although the overall number of blood glucose results was large enough to reach statistical significance, the number of results within each group with lower haematocrits was smaller. Therefore, the possibility of statistical type II error due to the limited sample size needs to be considered when interpreting the results comparing the different haematocrit groups.

Repeated blood glucose measurements were not performed on the same sample to assess the variability and repeatability of the results in both hand-held glucometers. Therefore, it remains uncertain whether this could have impacted the number of values falling within the allowable error for both hand-held glucose analysers.

Amongst all our results, there was only one that combined a low haematocrit (32%) and an off-range blood glucose result (12.1 mmol/L) based on the wet biochemistry analyser reference range. Due to this very limited sample size, we cannot extrapolate whether the presence of low haematocrit can affect the accuracy of hand-held glucometers in hypoglycaemic or hyperglycaemic dogs.

Operator variability, temperature, and humidity can also cause variability in glucose measurements when obtained by hand-held analysers (1, 2, 11). However, in our study, these were considered unlikely to have a significant effect on the variability between point-of-care glucometers and wet biochemistry, as all the samples were measured in a single referral hospital by only two trained, highly experienced laboratory technicians over the same season (winter).

## 5 Conclusion

The new models of point-of-care glucometers, G-PET PLUS^®^ and AlphaTrak3^®^, demonstrated a positive correlation with the reference analyser. Their performance was not adversely impacted in patients with low haematocrit compared to non-anaemic dogs, and can be used for clinical decision-making in anaemic, normoglycemic dogs when whole blood EDTA samples are tested within 1 h of collection. Clinicians should be mindful that whilst glucose readings in anaemic dogs typically varied from the reference method by only −0.82 mmol/L (−14.8 mg/dL) for the AlphaTrak3^®^, −0.17 mmol/L (−3.1 mg/dL) for the G-PET PLUS^®^, in some dogs the differences ranged from −1.2 mmol/L (21.6 mg/dL) to 2.0 mmoL/L (36 mg/dL). Therefore, whilst we suggest that immediate results from handheld glucometers can be used when rapid assessment is needed, serum glucose measurements are still recommended to confirm these results. We would also recommend that clinics take steps to evaluate the degree of bias for each glucometer they use in accordance with the ASVCP guidelines. Further studies are necessary to evaluate whether these results are different when using capillary blood or when glucose concentrations fall outside the reference range, particularly in the presence of anaemia.

## Data Availability

The datasets presented in this study can be found in online repositories. The names of the repository/repositories and accession number(s) can be found at: https://data.mendeley.com/datasets/y8p6nyxrtw/1.
